# Genetic diversity in Sickleweed (*Falcaria vulgaris*) and using stepwise regression to identify marker associated with traits

**DOI:** 10.1038/s41598-023-39419-5

**Published:** 2023-07-26

**Authors:** Mehdi Rahimi, Masoud AhmadiAfzadi, Mojtaba Kordrostami

**Affiliations:** 1 Department of Biotechnology, Institute of Science and High Technology and Environmental Sciences, Graduate University of Advanced Technology, Kerman, Iran; 2 Nuclear Science and Technology Research Institute (NSTRI), Nuclear Agriculture Research School, Karaj, Iran

**Keywords:** Plant biotechnology, Plant breeding, Plant ecology, Plant genetics

## Abstract

One of the well-known medicinal plants in the *Falcaria* genus is Sickleweed. *Falcaria* species exhibit a high degree of genetic variability, posing challenges in the examination of genetic diversity due to the significant potential for hybridization and introgression among them. Utilizing morphological traits and molecular markers may prove to be a valuable approach in evaluating and harnessing germplasm, considering the current obstacles faced in breeding this medicinal herb. In 2021, fifteen Sickleweed populations were cultivated in pots under field conditions, employing a randomized complete block design with three replications. This aimed to assess genetic diversity and conduct marker-trait association analyses utilizing morpho-physiological characteristics and SSR markers. The Sickleweed populations displayed considerable genetic diversity across all traits. Through cluster analysis of traits and the utilization of the UPGMA method based on the Gower distance matrix, the population was classified into three distinct clusters. Upon examining all genotypes, 52 polymorphic bands were detected, with an average of 8.68 bands per primer. The average expected heterozygosity across all loci was 0.864, while the average PIC was 0.855. Molecular data analysis employing the Jaccard similarity index and UPGMA method revealed the division of Sickleweed populations into two major groups. Furthermore, the results of molecular variance analysis indicated that variation within the population exceeded that between populations. Thirty-two SSR fragments were found to be significantly associated with genomic regions controlling the studied traits, determined through the application of stepwise regression. Selection based on molecular markers offers a rapid method for breeding programs, with the genetic information obtained from these markers playing a crucial role. Therefore, alongside traits, selecting superior genotypes and populations of high value in breeding programs becomes feasible. The findings highlight that certain markers are linked to multiple traits, emphasizing the critical importance of this characteristic in plant breeding for the simultaneous improvement of numerous traits. The study’s insights regarding markers hold potential for application in Sickleweed breeding programs.

## Introduction

Sickleweed (*F. vulgaris*), scientifically known as Falcaria, belongs to the Apiaceae family and is classified as a dicotyledonous plant. Its distinct leaves resemble the shape of a goose's foot, earning it various names such as Falcaria, Longleaf, and Sickleweed^1,2^. This species is found in different regions of Iran, including Tehran, Azarbaijan, Arak, Khorasan, Shirvan, Bojnord, Hamadan, Kurdistan, Kermanshah, and other areas. In some parts of Iran, it grows near fields and is consumed as a vegetable. Geographically, Sickleweed is distributed across America, Europe, Turkey, Iran, Caucasus, Central Asia, and Northwest Africa^3,4^. Sickleweed (*Falcaria* spp.) is an important plant with various uses and properties that make it worthy of investigation. It has been recognized for its medicinal value and is traditionally used in herbal medicine for its potential therapeutic effects. Sickleweed contains bioactive compounds that have shown antimicrobial, anti-inflammatory, antioxidant, and anticancer properties in previous studies^5^. Furthermore, Sickleweed possesses nutritional value and can be used as a valuable food source. It is rich in essential nutrients, vitamins, and minerals that contribute to human health and well-being. The exploration of the genetic diversity in Sickleweed is crucial for understanding its potential for breeding programs aimed at improving its medicinal and nutritional properties^6^. Traditionally, Sickleweed is commonly used both as a vegetable and as a medicinal plant, particularly during the spring season. Given the significance of vegetables and medicinal plants in human nutrition, it is essential to assess the plant's organic and mineral content^7,8^. Minerals like calcium and phosphorus play vital roles in bone formation, muscle contractions, and regulation of nervous excitability^9^. The pollination behavior of Sickleweed (*Falcaria vulgaris*) is an important aspect to consider in genetic diversity studies^10^. Sickleweed is a cross-pollinated species, meaning that it relies on the transfer of pollen between individual plants for successful fertilization^11^. In its natural habitat, Sickleweed is primarily pollinated by insects, including bees, butterflies, and other flying insects. These pollinators visit the flowers of Sickleweed to collect nectar and inadvertently transfer pollen between flowers, facilitating cross-pollination^10^. Understanding the pollination behavior of Sickleweed is crucial for several reasons^12^. First, it influences the genetic composition of the populations, as cross-pollination leads to genetic recombination and increases genetic diversity. Second, it affects the distribution of genetic traits within and among populations, as specific pollinators may preferentially visit certain individuals or populations. Finally, it has implications for the conservation and management of Sickleweed populations, as changes in pollinator availability or behavior can impact reproductive success and genetic diversity.

Plant breeding relies on diversity and selection, with genetic diversity playing a crucial role in the visibility of breeding activities and the selection of suitable candidates for breeding programs^13,14^. The foundation of diversity in any plant species lies within its genetic resources, particularly ecotypes and wild populations, which serve as valuable components for breeding programs^15^. Therefore, the initial step involves the collection of ecotypes and diverse populations from different regions to assess their genetic diversity. This process necessitates a meticulous examination of the genetic diversity within the germplasm^13,15^. Awareness of the extent of variation and the relationships among traits, including their correlation with important and economically significant traits such as yield, forms the basis for efficient and precise identification and selection of parent plants^16^. Estimating genetic diversity in plants is crucial for the development of effective breeding programs and the preservation of genetic resources. To achieve increased production and improved quality of plant products, as well as the optimal utilization of genetic resources, the collection, storage, description, and evaluation of genetic materials are imperative^17^. Initially, evaluations of diversity were primarily based on morphological assessments encompassing morphological, physiological, agronomic, and biochemical traits. These morphological evaluations continue to be widely used due to their simplicity and suitability for specific research goals and desired levels of accuracy. While morphological diversity can be influenced by environmental conditions to a greater extent compared to molecular diversity, it remains a valuable tool across various plant species. Identifying morphological diversity not only aids in the management of plant germplasms but also provides valuable insights to researchers in the field of plant breeding^18,19^.

In recent years, there has been a notable surge in the utilization of molecular tools for investigating genetic diversity, conducting QTL mapping, and implementing protective programs in plants and other organisms. The information derived from these genetic techniques serves as a valuable parameter for studying diverse populations and comprehending genetic distinctions between them^20,21^. Among the most effective markers for analyzing genetic diversity and identifying allelic variations at specific loci are SSR (Simple Sequence Repeat) molecular markers, known for their high diversity value^22^. SSR markers have been extensively employed in diverse plant species, including medicinal plants, to assess genetic diversity^23–27^. These markers have demonstrated high efficiency in such studies. However, there has been limited research conducted on Sickleweed plants using SSR markers. In the study of the genetic diversity of the this population with SCoT molecular marker and morphological traits under different geographical and climatic conditions, the population was divided into three and two groups based on the markers and traits, respectively^28^. Also, another study by Piya et al.^29^ stands as the sole investigation in this regard. The study employed SSR markers to analyze the genetic diversity of eight distinct Sickleweed populations, each comprising 12 individuals sourced from various locations across the United States. The findings revealed the division of these individuals into three groups based on SSR markers, indicating significant genetic diversity among them.

An essential aspect of plant breeding programs involves studying the correlation between DNA polymorphism and phenotypic trait diversity^30^. This investigation holds numerous applications, including the examination of phylogenetic relationships among genotypes and unknown populations, identification of desirable trait alleles in germplasm collections, precise localization of quantitative trait loci, and confirmation of candidate genes associated with quantitative traits. The identification of genomic regions governing quantitative traits relies on linkage disequilibrium and is achieved through two main approaches: linkage analysis and association analysis^31^. In association analysis, the direct examination of the relationship between genotype and phenotype in individuals is employed to identify chromosomal regions involved in trait control^32^. Marker-trait association analysis has been extensively employed in various plant species^33^, including medicinal plants^34–40^. However, there have been limited studies investigating genetic diversity at the phenotypic and molecular levels, as well as the associations between molecular markers and phenotypes, in Sickleweed. Consequently, this research aims to explore the genetic diversity of Sickleweed ecotypes collected from different regions using SSR markers and traits, while also conducting association analyses between traits and markers. Undoubtedly, the findings of this study will provide valuable insights for further research and breeding programs focused on this medicinal and vegetable plant. By studying the genetic diversity of Sickleweed, we can identify and characterize different genotypes with distinct traits and determine their potential for cultivation, selection, and breeding purposes. This knowledge can facilitate the development of improved varieties with enhanced medicinal and nutritional qualities, as well as better adaptability to different environments.

## Materials and methods

This study was conducted in compliance with the relevant institutional, national, and international guidelines and legislation of Iran. No specific permits were required for the collection of plant materials. The seeds of 15 different populations of Sickleweed were collected from seven Iranian provinces, namely Ardebil, Kurdistan, Kermanshah, Gilan, Hamedan, Qazvin, and Qom (Table 1). The pollination behavior of Sickleweed (*Falcaria vulgaris*) is an important factor to consider in studies assessing genetic diversity. Sickleweed is primarily a cross-pollinated species, relying on the transfer of pollen between individual plants for successful fertilization. The specific pollination behavior, including the involvement of different pollinators and the extent of self-pollination, can have significant implications for the composition and diversity of Sickleweed populations. It is important to consider pollination behavior when collecting samples for genetic diversity studies. To ensure representative sampling and capture the genetic diversity within populations, it is crucial to employ a strategy that accounts for potential differences in pollination patterns among individuals and populations. In our study, we followed a systematic approach for sample collection, taking into consideration the pollination behavior of Sickleweed. We employed a random sampling strategy, ensuring that individuals were selected from different locations within each population. By collecting samples from multiple individuals within a population, we aimed to capture the genetic variation present within and among populations, including potential variations resulting from pollination patterns. Furthermore, we acknowledge the importance of providing detailed information on the sample collection strategy, including the number of plants sampled per population, the collection locations, and any additional considerations specific to pollination behavior. We will revise the manuscript to include these details and emphasize the importance of incorporating the pollination behavior in the sampling strategy to obtain a comprehensive assessment of genetic diversity in Sickleweed populations. The species identity was confirmed by Dr. Rahimi, and voucher specimens were deposited in the Graduate University of Advanced Technology Herbarium under the numbers 151 to 165. These specimens are available for botanical studies upon official request. The 15 different populations of Sickleweed were cultivated in pots under field conditions in 2021 at the Graduate University of Advanced Technology in Kerman, Iran. The plants were grown in pots located in the field and field conditions. The growth media used in this study consisted of a well-balanced mixture of organic and inorganic components to provide optimal nutrient availability and support healthy plant growth. The growth media composition consisted of a combination of sterile soil, peat moss, and perlite in a ratio of 3:1:1. This mixture was selected based on previous studies and recommendations for the cultivation of Sickleweed to provide a suitable substrate for plant growth. The sterile soil provided essential minerals and nutrients, while the peat moss and perlite contributed to the media's moisture retention and aeration properties. To ensure the consistency of growth conditions, the growth media were thoroughly mixed and sterilized before planting the Sickleweed seeds. The pots were filled with the growth media, and the seeds were sown at the recommended depth. The experimental design employed was a randomized complete block design with three replications. In our study, we maintained a single Sickleweed plant per pot to minimize competition and facilitate accurate data collection. This allowed us to focus on the individual performance of each plant and avoid potential confounding effects. To represent the genetic diversity within Sickleweed populations, we used multiple pots for each population. The number of samples for each pot in each replicate was 10, and their average was used for the data of each replicate. To ensure the reliability of our results, we incorporated 3 replications in our experimental design. The number of replications varied depending on the specific analysis and statistical requirements of each trait. We employed a sufficient number of replications to minimize the impact of random variability and enhance the statistical power of our findings. The specific details regarding the number of plants per pot, the number of pots per population, and the number of replications were carefully considered during the planning and execution of our study. These design considerations aimed to ensure the validity and robustness of our results and provide meaningful insights into the genetic diversity and marker-trait associations in Sickleweed. During the vegetative stage, various morphological traits such as plant height, fresh and dry weight, leaf number, length, and width were measured. After harvesting, the leaves of the plants were collected, wrapped in foil, and transported to the laboratory for DNA extraction and assessment of nutrient content. The nutrient content analysis focused on elements such as zinc, manganese, potassium, iron, sodium, magnesium, and calcium ions. These measurements were carried out using flame and atomic absorption methods. The GTA-110 graphite tube atomizer Spectra AA 220Varian, manufactured in Australia, was utilized for the measurement of dissolved ions^41^. Before sample measurement, the standard solution of each ion was injected into the device, and the corresponding standard curve was generated using the device's software (Spectra AA). The unknown concentrations of the solutions were determined using software^42^.

**Table 1:** Characteristics of the collection areas of Sickleweed plant samples.

Herbarium number	Province	City	Region	Longitude	Latitude	Height above sea level
151	Kermanshah	Sonqor	Kivananat	47.27889	34.84972	1847
152	Kermanshah	Sonqor	Bavaleh	47.69778	35.02722	2010
153	Kermanshah	Sahneh	Sahneh	47.75833	34.44194	1465
154	Hamedan	Asad abad	Chaharduli	48.065	34.93139	1890
155	Kurdistan	Gorveh	Panjeh Ali	47.70834	35.18667	1912
156	Kurdistan	Dehgolan	Bolbanabad	47.41195	35.15222	1851
157	Kurdistan	Dehgolan	Amirabad	47.31084	35.10361	1974
158	Kurdistan	Kamyaran	Gerger-e Sofla	47.27667	35.00334	1824
159	Kurdistan	Kamyaran	Qaleh Gah	47.21196	34.95001	1611
160	Kurdistan	Bijar	Nemat abad auliya	47.41333	35.68444	1922
161	Kurdistan	Bijar	Seylatan	47.83054	36.03666	1617
162	Qazvin	Qazvin	Ilan	50.64778	36.42528	1450
163	Ardabil	Meshkinshahr	Shaban	47.44833	38.37556	1236
164	Qom	Qom	Khalajastan	50.19028	34.67944	1952
165	Gilan	Siahkal	Deylaman	49.90528	36.88889	1455

To extract nutrient content from the plant tissue, the following procedure was followed. A dry plant tissue sample weighing 0.5 g was placed in 10 ml of concentrated nitric acid (65%) and allowed to dissolve in the acid for 24 h. After this period, the sample was heated to release acid vapors. Distilled water was then added to bring the solution volume up to 50 ml. The solution was filtered through filter paper to remove any particulate matter and obtain a clear solution suitable for analysis in an atomic absorption device. In addition to the atomic absorption analysis, the nitrogen percentage in the plant tissue was measured and calculated using the Kjeldahl method. This involved a process of digestion, distillation, and titration using a Kjeldahl apparatus.

Descriptive statistics, including measures such as mean, range, and standard deviation, were computed for the average data of the 15 populations. Additionally, the phenotypic coefficient of variation (PCV) was calculated for the studied traits. The PCV is a measure of variation relative to the mean and is often expressed as a percentage. To calculate the PCV for a trait, the following formula can be used:

$${\mathrm{CV}}_{\mathrm{p}}=\left(\sqrt{{\upsigma }_{\mathrm{p}}^{2}}/\overline{\mathrm{x} }\right)\times 100$$

The PCV provides insights into the relative magnitude of variation for a specific trait compared to its mean value. It helps assess the extent of phenotypic diversity within the populations under study.

The analysis of variance (ANOVA) was conducted using the randomized complete block design, and the expected value of the mean square was used to determine the variance components. To analyze the zinc and manganese features, which had small measured data sizes, the data were initially multiplied by 100 before undergoing the analysis of variance. The software used for performing the analysis of variance was SAS 9.4^43^. In the laboratory of the Graduate University of Advanced Technology in Kerman, Iran, DNA extraction was carried out from young leaf samples of Sickleweed ecotypes using the Dellaporta method^44^ with some modifications. After DNA extraction, the quality of the samples was assessed through 2% agarose gel electrophoresis, while the quantity of DNA was determined using spectrophotometry. The polymerase chain reaction (PCR) was conducted using six microsatellite primers that had been previously investigated for their suitability in Sickleweed^2^.

For the SSR marker, the polymerase chain reaction (PCR) was carried out in a volume of 10 µl. The reaction mixture included 2 µl of template DNA (50 ng), 0.6 µl of dNTP (10 mM), 0.3 µl of MgCl_2_ (50 mM), 0.12 µl of *Taq* polymerase enzyme (5U), 1 µl of PCR buffer (10×), 0.4 µl of the forward primer (60 ng), 0.4 µl of the reverse primer (60 ng), and 5 µl of sterile deionized water. The PCR thermal cycle for the microsatellite marker consisted of an initial denaturation step at 94 °C for 4 min, followed by 35 cycles. Each cycle included a 30-s denaturation step at 94 °C, a 45-s annealing step at the temperature indicated by the primer's melting temperature (TM) in Table 4, a 2-min extension step at 72 °C, and a final extension step at 72 °C for 5 min. The reaction mixture was then cooled to 4 °C. The PCR products were detected using a Bio-Rad Sequi-Gen vertical electrophoresis machine and a 6% polyacrylamide gel. The staining method described by An et al.^45^ was employed with some modifications (Table 4).

The banding patterns obtained from the PCR analysis were scored as either absence (zero) or presence (one) of a band. These scores were organized in a matrix format with populations as rows and bands as columns. This matrix was used to calculate various marker indexes and to group the populations accordingly.

Marker indexes were calculated using specific formulas and a custom Excel program developed by the first author. These indexes included the number of amplified bands, the number of polymorphic bands, the polymorphic percentage, Polymorphism Information Content (PIC)^46^, Expected Heterozygosity^47^, Marker Index, Effective Multiplex Ratio and Mean Heterozygosity^48^, and Marker Detection Power^49^. Furthermore, the number of effective alleles, Shannon's index^50^, and Nei's genetic diversity^51^ were determined using the POPGEN software version 1.3.1^52^. Cluster analysis was performed using different methods and criteria based on either the studied traits or SSR markers to group the ecotypes. The cluster method and criterion that yielded the highest Cophenetic correlation coefficient were selected, and the grouping was performed using the chosen method with the R software. The determination of the number of groups was based on the maximum distance method of merging two groups. The analysis of molecular variance (AMOVA) was conducted using the GenAlex software package version 6.5^53^. This analysis partitioned the total molecular variance among and within all populations based on the number of groups obtained from the cluster analysis. To examine the relationship between the molecular data (independent variables) and the quantitative data (dependent variables), stepwise regression analysis was performed. The stepwise regression utilized a step-by-step method and was conducted using the PAST software^54^.

### Ethics approval and consent to participate

This study complied with relevant institutional, national, and international guidelines and legislation of Iran, and no specific permits were required to collect the plant materials. The species identity was done by Dr. Rahimi and voucher specimens were deposited in the Graduate University of Advanced Technology Herbarium (no. 151 to 165), available for botanical studies with an official request.

## Results

Descriptive statistics, such as the minimum, maximum, and range of the studied traits, are presented in Table 2. The analysis of the Phenotypic Coefficient of Variation (PCV) for these traits reveals a favorable level of variation, indicating their potential utility in enhancing the studied populations of Sickleweed (Table 2). Notably, Potassium exhibits the lowest PCV value of 5.75, while Leaf width demonstrates the highest PCV of 38.50, followed by Zinc with a PCV of 34.30. The remaining traits fall within the range of PCV values (Table 2).

**Table 2:** Descriptive statistics of morphological and elements traits in Sickleweed populations.

Traits	Statistical parameters					
	Min	Max	Range	Avereage	Stand. dev	PCV
Leaf number (number)	10.93	18.84	7.91	14.69	2.35	15.97
Leaf length (mm)	17.54	37.92	20.38	25.98	5.52	21.24
Leaf width (mm)	1.13	4.57	3.44	2.69	1.04	38.50
Plant height (cm)	39.60	60.03	20.43	48.67	5.94	12.20
Fresh weight (gr)	18.37	32.62	14.25	25.55	4.83	18.91
Dry weight (gr)	4.60	9.20	4.60	6.55	1.53	23.38
Zinc (mg/g)	22.91	74.77	51.86	50.10	17.19	34.30
Manganese (mg/g)	4.20	8.42	4.22	6.53	1.30	19.95
Potassium (mg/g)	621.02	766.71	145.68	704.07	40.47	5.75
Iron (mg/g)	25.14	36.15	11.00	30.87	3.46	11.21
Sodium (mg/g)	33.95	48.22	14.28	39.95	4.34	10.86
Magnesium (mg/g)	13.02	24.02	11.00	18.10	3.53	19.49
Calcium (mg/g)	1.27	3.44	2.18	2.33	0.66	28.14
Nitrogen (%)	20.02	38.30	18.27	27.96	5.56	19.88

The analysis of variance results for the studied traits, including leaf characteristics and elements, indicated a significant difference (p < 0.01) among the Sickleweed populations (Table 3). This significant difference highlights the presence of notable diversity among these populations.

**Table 3:** The analysis of variance for morphological and elements traits in Sickleweed populations based on randomized complete block design.

	Df.	Sources of variation			CV of design (%)
		R	Populations	Error	
		2	14	28	
The mean square of traits	Nitrogen	0.66 ns	92.69**	0.48	2.48
	Calcium	0.016 ns	1.29**	0.043	8.9
	Magnesium	0.14 ns	37.31**	0.13	2.01
	Sodium	0.028 ns	56.43**	0.17	1.04
	Iron	0.31 ns	35.91**	0.37	1.97
	Potassium	15.88 ns	4912.98**	5.46	0.33
	Manganese	0.0001 ns	5.09**	0.0009	0.46
	Zinc	0.046 ns	886.16**	0.14	0.74
	Dry weight	0.069 ns	7.03**	0.75	13.22
	Fresh weight	15.72 ns	70.08**	15.25	15.28
	Plant height	11.16 ns	105.69*	48.08	14.24
	Leaf width	0.006 ns	3.22**	0.0065	3.001
	Leaf length	2.99 ns	91.36**	2.22	5.73
	Leaf number	0.64 ns	16.51**	0.45	4.59

^ns^, * ^and^ **: Non-Significant and significant at 5% and 1% probability levels, respectively.

Table 4 presents the results of the variance components, ranging from 28.5 to 99.5%. These findings indicate a significant amount of variation between the populations, which can be utilized in the selection of superior populations. Additionally, variation within the populations was observed for certain traits, ranging from 0.05 to 71.46%. This internal variation offers the opportunity to select the best individuals within each population, particularly for traits such as Plant height and Fresh weight, which exhibit high levels of diversity.

**Table 4:** Estimation of the variance components of the sources of variation in the unbalanced nest design of the studied physiological and biochemical traits.

Traits	Sources of variation			
	Populations		Error	
	Variance components	%	Variance Components	%
Leaf number	5.35	92.25	0.45	7.75
Leaf length	29.71	93.05	2.22	6.95
Leaf width	1.07	99.40	0.0065	0.60
Plant height	19.20	28.54	48.08	71.46
Fresh weight	18.28	54.51	15.25	45.49
Dry weight	2.09	73.62	0.75	26.38
Zinc	295.34	99.95	0.14	0.05
Manganese	1.70	99.95	0.0009	0.05
Potassium	1635.84	99.67	5.46	0.33
Iron	11.85	96.97	0.37	3.03
Sodium	18.75	99.10	0.17	0.90
Magnesium	12.39	98.96	0.13	1.04
Calcium	0.42	90.63	0.043	9.38
Nitrogen	30.74	98.46	0.48	1.54

**Figure 1:**
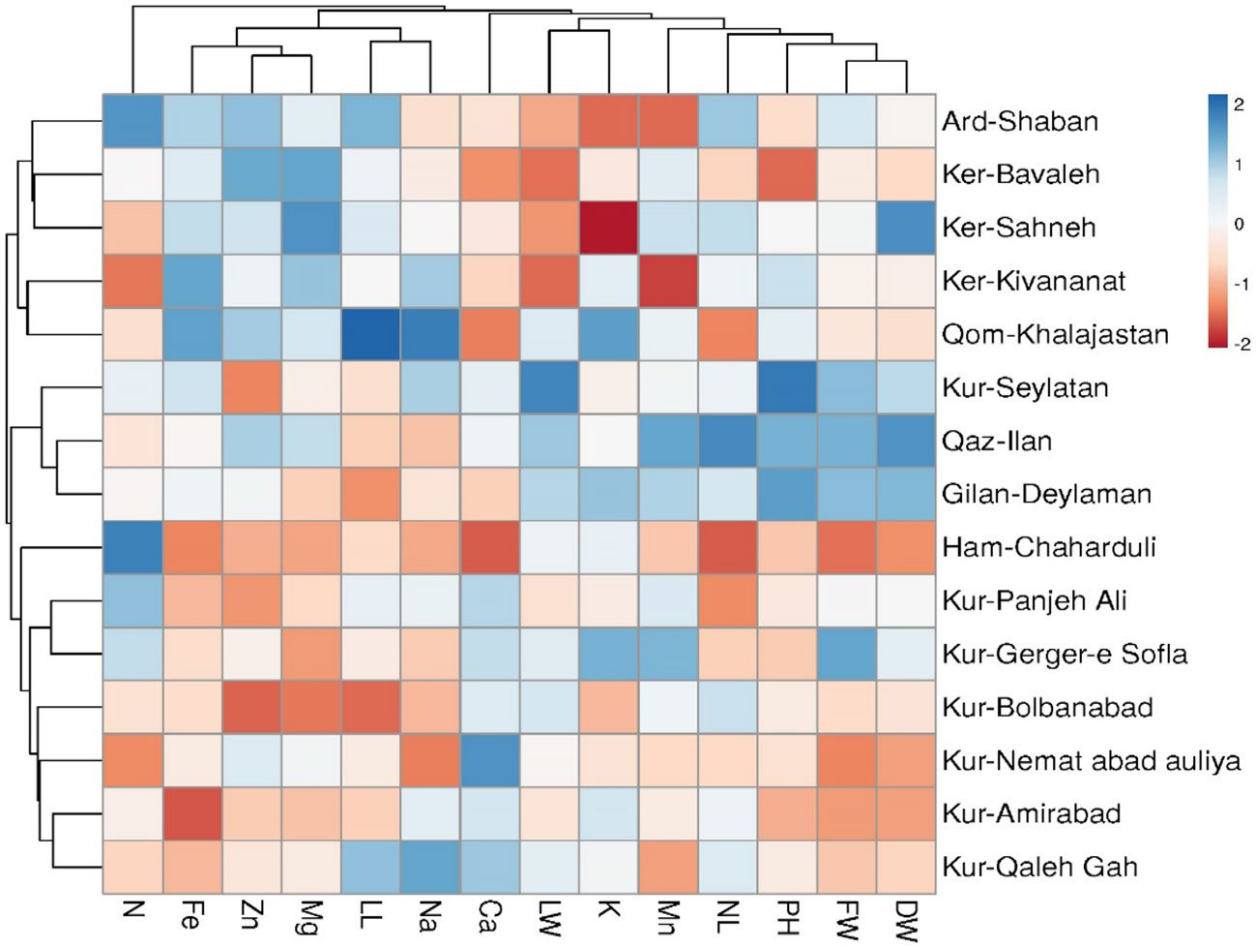
The UPGMA cluster analysis of the Sickleweed population based on morphological and elements traits.

The UPGMA method with Gower distance yielded the highest Cophenetic correlation coefficient value (0.756) among all the methods using different distance criteria. Consequently, cluster analysis was performed using this method, resulting in the division of the Sickleweed populations into three distinct groups (Fig. 1). The genetic distances between populations varied, with the highest genetic distance (0.586) observed between the Ker-Sahneh and Ham-Chaharduli populations, and the lowest genetic distance (0.184) observed between the Qaz-Ilan and Gilan-Deylaman populations. As depicted in Fig. 1, the Sickleweed populations were categorized into three main groups. The first group included the populations of Ker-Kivananat, Ker-Bavaleh, Ker-Sahneh, Ard-Shaban, and Qom-Khalajastan. The second group consisted of Kur-Seylatan, Qaz-Ilan, and Gilan-Deylaman populations. The third group comprised the populations of Ham-Chaharduli, Kur-Panjeh Ali, Kur-Bolbanabad, Kur-Amirabad, Kur-Gerger-e Sofla, Kur-Qaleh Gah, and Kur-Nemat abad auliya. It is worth noting that the classification of distinct populations into different groups may be attributed to variations in genetic backgrounds or other environmental factors. Physical characteristics may serve as a basis for this classification. The results of the cluster analysis provide insights into the differences, relationships, and similarities among the populations within each group. The genetic composition or environmental influences may account for the observed differences between these groups.

In this study, six SSR primers were used, resulting in a total of 55 bands. Among these bands, 52 were polymorphic, with an average of 8.67 polymorphic bands per primer (Table 5). The GSSR24 primer exhibited the highest number of bands, with 13 bands, while the ESSR80 primer had the lowest number, with four bands (Table 5). The percentage of polymorphism observed in the Sickleweed populations varied from 81.82 to 100% across the different primers, with an average percentage of polymorphism of 94.59% (Table 5). The polymorphic information content (PIC) ranged from 0.705 to 0.926, with an average PIC of 0.855 (Table 5). The expected heterozygosity (H) for the SSR markers ranged from 0.729 to 0.928, with an average expected heterozygosity of 0.864 (Table 5). The marker index (MI) varied among the SSR markers, with the ESSR80 primer exhibiting the highest MI (0.136) and the GSSR24 primer showing the lowest (0.020) (Table 5). The average heterozygosity (Havp) ranged from 0.0042 to 0.0678 for the SSR markers. The ESSR80 and BSSR53 primers had the highest average heterozygosity, indicating their high efficiency in detecting polymorphism (Table 5). Assessing genetic diversity among cultivars and populations often involves evaluating the genetic diversity index. Nei's gene diversity index ranged from 0.370 to 0.458 among the SSR primers, with an average of 0.414 in the studied population (Table 5). The ESSR80 and GSSR25 primers exhibited the highest genetic diversity, respectively. The mean Shannon's coefficient for the SSR markers was 0.601, indicating an average level of diversity within the investigated populations. The ESSR80 and GSSR25 primers had the highest values of Shannon's index, suggesting that these primers captured a greater extent of genetic diversity within the population (Table 5). The number of effective alleles ranged from 1.632 to 1.856, with an average of 1.734 in the studied population (Table 5).

**Table 5:** Calculated indices of molecular markers for SSR primers.

Indices^a^	Primers						Average
	ESSR9	BSSR53	GSSR154	GSSR24	ESSR80	GSSR25	
TB	11	8	12	13	4	7	9.17
PB	9	8	12	13	4	6	8.68
PP	81.82	100	100	100	100	85.71	94.59
PIC	0.853	0.852	0.898	0.926	0.705	0.898	0.855
EH	0.86	0.86	0.902	0.928	0.729	0.902	0.864
D	0.921	0.921	0.967	0.995	0.781	0.966	0.925
MI	0.045	0.047	0.035	0.02	0.136	0.035	0.053
EMR	2.867	2.667	4.333	4.8	2	3.933	3.433
Havp	0.0156	0.0175	0.0081	0.0042	0.0678	0.0089	0.02
NEA	1.632	1.741	1.729	1.638	1.856	1.809	1.734
SI	0.567	0.606	0.606	0.549	0.65	0.629	0.601
NGD	0.381	0.418	0.416	0.37	0.458	0.44	0.414

^a^Total amplified bands (TB), Number of polymorphic bands (PB), Polymorphic percentage (PP), Polymorphism Information Content (PIC), Expected Heterozygosity (EH), Marker detection power (D), Marker Index (MI), Effective Multiplex Ratio (EMR), Mean Heterozygosity (Havp), Number of effective alleles (NEA), Shannon's index (SI) and Nei's genetic diversity (NGD).

**Figure 2:**
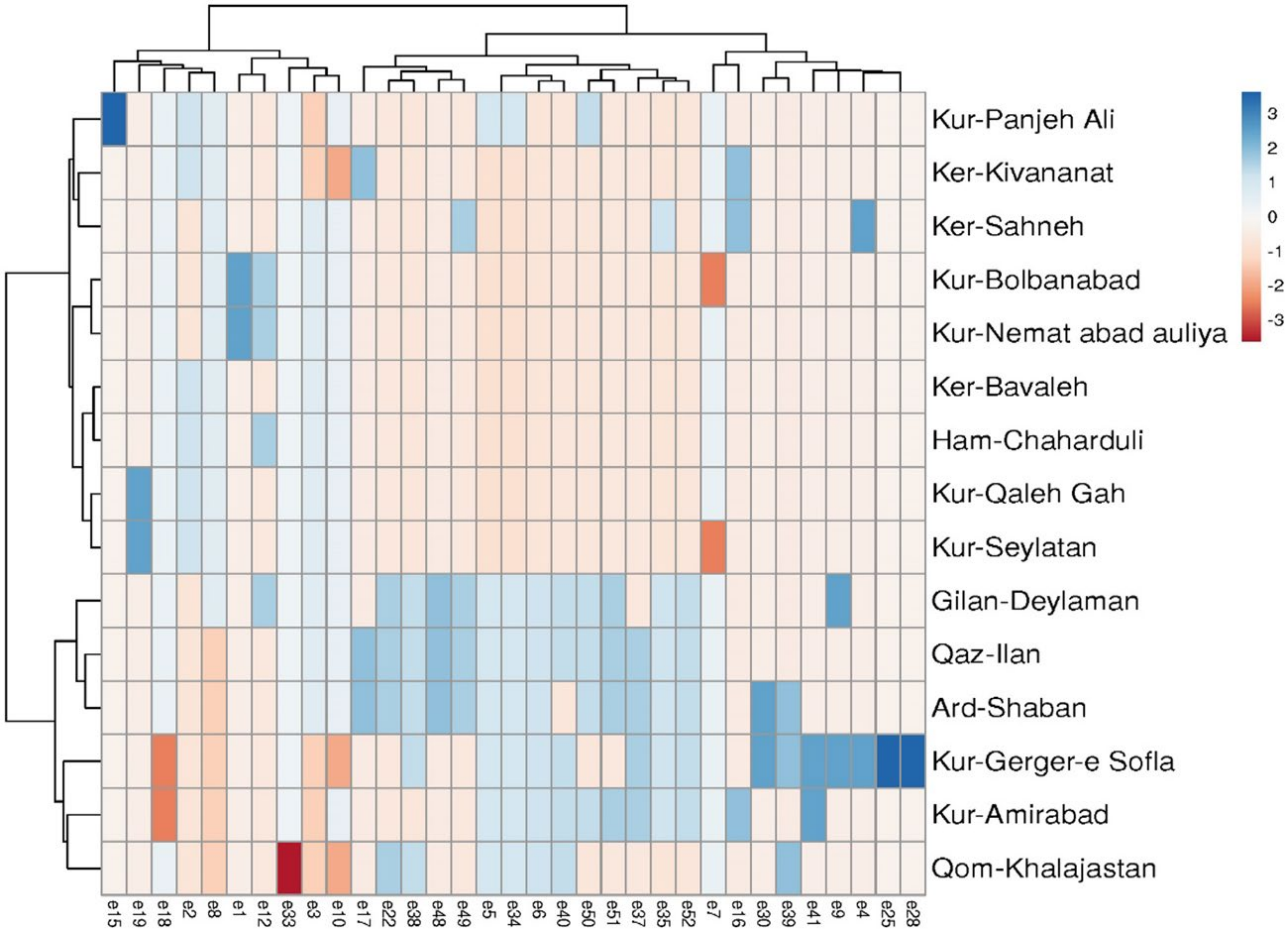
Dendrogram resulting from cluster analysis of Sickleweed populations with UPGMA and Jaccard similarity index based on SSR markers.

The evaluation of different methods of cluster analysis using various similarity criteria revealed that the UPGMA method with the Jaccard similarity index demonstrated the highest Cophenetic correlation coefficient value (0.62). Figure 2 illustrates the clustering results obtained using this method. The Sickleweed populations were divided into three distinct groups based on the molecular data, which aligned closely with the phenotypic data. The populations of Kur-Seylatan and Ard-Shaban exhibited the lowest genetic distance (0.0625), indicating a high level of genetic similarity. On the other hand, the populations of Ham-Chaharduli and Ard-Shaban displayed the highest genetic similarity (0.52). As depicted in Fig. 2, the studied populations were categorized into two groups. The first group comprised Qom-Khaljastan, Kur-Amirabad, Kur-Gerger-e Sofla, Ard-Shaban, Qaz-Ilan, and Gilan-Deylaman.The second group consisted of Kur-Panjeh Ali, Ker-Kivananat, Ker-Sahneh, Kur-Bolbanabad, Kur-Nemat abad auliya, Ker-Bavaleh, Ham-Chaharduli, Kur-Qaleh Gah, and Kur-Seylatan populations. The clustering results highlight the similarities and differences between the populations within each group. The observed genetic distances or similarities may be attributed to varying genetic compositions or other environmental factors influencing the traits studied.

To understand the differentiation of subgroups, molecular variance analysis was carried out with the assumption of three groups and the results showed that 18% of the total molecular diversity is explained by diversity among the populations, and 82% of that variation is related to within populations (Table 6).

**Table 6:** Analysis of molecular variance based on SSR markers.

Source	df	SS	MS	Est. Var.	% variance
Among Pops	1	13.811	13.811	1.184	18%
Within Pops	13	68.722	5.286	5.286	82%
Total	14	82.533		6.470	100%
PhiPT value			0.183		
PhiPT Prob			0.030		

In this study, a total of 49 markers (alleles) were found to have a significant relationship with the studied traits and were included in the regression model (Table 7). Some of these markers were found to have an impact on multiple traits, resulting in a final selection of 32 markers that effectively explained the phenotypic variations of these traits. These markers can be valuable in identifying superior genotypes based on the studied traits. However, it should be noted that other markers did not show a significant effect on the model. The number of identified markers ranged from one for Leaf number and Leaf width traits to nine for Potassium. These markers exhibited either positive or negative correlations with the studied traits. Notably, no marker showed a significant relationship with the Fresh weight trait. The proportion of phenotypic variation (R^2^) explained by each marker for the studied traits is presented in Table 7, with values ranging from 31 to 100% for the different traits. For instance, the marker GSSR24-3 was associated with the Leaf number trait and explained 46% of its phenotypic variation. Similarly, the marker GSSR24-13 showed a significant relationship with Leaf width, explaining 31% of its phenotypic variation. Furthermore, two markers, GSSR24-12 and ESSR80-4, were correlated with the Leaf length trait and collectively accounted for 50% of its variation. By calculating the standardized β values, it was determined that GSSR24-12 had greater importance and exerted a reducing effect on the trait (Table 7).

**Table 7:** Stepwise regression analysis of studied traits (dependent variable) and SSR markers (independent variables) in Sickleweed.

Trait	Marker	R	R^2^	E	R^2^ change	F of R^2^ change	Standardized beta coefficients	t-value
Leaf number	GSSR24-3	0.68	0.46	1.78	0.46	11.24**	0.68	3.35**
Leaf length	GSSR24-12	0.53	0.28	4.86	0.28	5.03*	− 0.655	− 3.09**
	ESSR80-4	0.71	0.50	4.22	0.22	5.26*	− 0.486	− 2.29*
Leaf width	GSSR24-13	0.56	0.31	0.89	0.31	5.9*	− 0.559	− 2.43*
Plant height	ESSR80-3	0.72	0.52	4.23	0.52	14.32**	− 0.956	− 6.86**
	ESSR9-5	0.84	0.71	3.44	0.19	7.79*	0.425	3.15**
	ESSR9-7	0.91	0.82	2.85	0.11	6.57*	0.346	2.56*
Dry weight	GSSR25-5	0.55	0.298	1.33	0.298	5.53*	1.46	41.91**
	GSSR24-4	0.72	0.519	1.15	0.22	5.49*	− 1.21	− 39.6**
	GSSR154-1	0.88	0.77	0.83	0.251	12**	− 0.71	− 29.7**
	GSSR154-5	0.94	0.884	0.62	0.114	9.77*	0.57	18.7**
	ESSR80-3	0.97	0.948	0.43	0.065	11.29**	− 0.29	− 13.7**
	BSSR53-6	0.997	0.995	0.15	0.046	69.83**	− 0.32	− 11.3**
	GSSR154-3	0.999	0.998	0.11	0.003	8.37*	− 0.08	− 2.89*
Zinc	GSSR24-12	0.729	0.532	12.2	0.532	14.77**	− 0.569	− 20.3**
	GSSR154-1	0.86	0.739	9.47	0.208	9.55**	0.344	13.7**
	ESSR9-1	0.942	0.887	6.5	0.148	14.46**	0.442	14.5**
	ESSR9-7	0.975	0.95	4.56	0.062	12.38**	− 0.289	− 11.4**
	GSSR25-1	0.99	0.98	3.02	0.03	13.8**	− 0.194	− 7.7**
	ESSR9-5	0.996	0.992	2.04	0.012	11.79**	0.163	5.5**
	GSSR24-3	0.998	0.996	1.49	0.004	8.01*	− 0.099	− 2.8*
Manganese	GSSR154-7	0.605	0.366	1.07	0.366	7.5*	− 0.904	− 5.5**
	GSSR25-4	0.784	0.625	0.87	0.249	7.7*	− 0.826	− 4.3**
	ESSR80-3	0.876	0.768	0.71	0.153	7.2*	− 0.491	− 2.7*
Potassium	BSSR53-1	0.689	0.475	30.4	0.475	11.7**	− 0.791	− 48.8**
	GSSR25-6	0.872	0.76	21.4	0.284	14.1**	0.797	45.8**
	BSSR53-2	0.943	0.889	15.2	0.129	12.8**	− 0.63	− 28.6**
	GSSR24-13	0.968	0.936	12.1	0.047	7.3*	− 0.137	− 7.1**
	GSSR24-10	0.985	0.971	8.6	0.035	10.6*	0.375	18.3**
	ESSR80-4	0.991	0.983	6.9	0.012	5.8*	− 0.298	− 12.4**
	ESSR9-5	0.996	0.992	5.1	0.009	7.7*	0.107	6.2**
	BSSR53-5	0.998	0.997	3.5	0.005	8.7*	− 0.169	− 6.6**
	GSSR25-4	1	0.999	1.7	0.003	20.1**	− 0.074	− 4.5**
Iron	BSSR53-7	0.658	0.433	2.7	0.433	9.9**	0.598	5.6**
	BSSR53-1	0.82	0.672	2.1	0.239	8.7*	0.649	6.5**
	GSSR24-1	0.894	0.799	1.7	0.127	6.9*	0.804	5.4**
	GSSR154-4	0.958	0.917	1.2	0.118	14.3**	− 0.507	− 3.7**
Sodium	ESSR9-3	0.632	0.399	3.4	0.399	8.6*	0.60	3.4**
	GSSR24-5	0.79	0.624	2.8	0.225	7.1*	− 0.475	− 2.6*
Magnesium	GSSR24-8	0.621	0.386	2.9	0.386	8.2*	0.859	6.4**
	BSSR53-2	0.834	0.695	2.1	0.31	12.2**	− 0.793	− 5.2**
	ESSR80-4	0.907	0.822	1.7	0.127	7.9*	− 0.413	− 2.8*
Calcium	GSSR24-8	0.576	0.331	0.55	0.331	6.4*	− 0.38	− 2.6*
	GSSR24-1	0.778	0.605	0.45	0.274	8.3*	− 0.807	− 5.3**
	GSSR25-3	0.864	0.746	0.37	0.141	6.1*	− 0.582	− 3.6**
	ESSR9-8	0.914	0.836	0.31	0.09	5.5*	0.348	2.3*
Nitrogen	BSSR53-5	0.56	0.314	4.7	0.314	5.9*	− 0.485	− 2.6*
	BSSR53-6	0.793	0.629	3.6	0.315	10.2**	− 0.688	− 4.2**
	GSSR154-11	0.867	0.752	3.1	0.123	5.5*	0.456	2.3*

* and **: Significant at 5% and 1% probability levels, respectively.

## Discussion

The high phenotypic coefficient of variation (PCV) observed for Leaf width and Zinc traits indicates that these traits have a greater potential for improvement and modification through selective breeding programs. This suggests that there is a higher chance of selecting superior populations among the studied Sickleweed populations for these traits. Conversely, the Potassium trait exhibits the lowest PCV, implying that improving this trait through selection in the studied population may be less successful compared to other traits. The observed high diversity in traits among the Sickleweed populations can be attributed to environmental conditions as well as the genetic variations among populations. The phenotypic evaluations and dispersion indices demonstrate that the studied populations exhibit significant diversity across various traits. This diversity can be valuable for association analysis and breeding programs. Furthermore, the results of variance analysis highlight the existence of inherent genetic diversity among the studied populations across all traits. This emphasizes the possibility of identifying superior populations or genotypes based on desired traits. Similar findings have been reported by researchers studying various medicinal plants, highlighting the potential to utilize phenotypic diversity in populations or genotypes for achieving superior populations or genotypes and managing breeding programs based on them. These traits have proven effective in identifying and characterizing diversity within populations^55–59^.

The variance component analysis revealed that, except for the Plant height trait, the variation between populations was greater than within populations for all other traits. This difference in variation can be attributed to the diverse environmental and genetic conditions among the populations. Morphological traits are known to be influenced by the growth environment, and the existing variations in environmental and growth conditions contribute to the observed diversity in these traits. It is important to note that morphological traits, being polygenic, may not accurately reflect genetic changes at the genomic level. Therefore, grouping populations based on these traits may yield different results and changes under different environmental conditions. This emphasizes the need for careful consideration when using morphological traits alone for grouping or selection purposes^55,56^. To maximize heterosis, it is beneficial to cross genotypes or cultivars that have significant genetic dissimilarity^60^. Phenotypic traits can be used as indicators to select parents with a substantial genetic distance between genotypes for such crosses. Multivariate analysis techniques, such as cluster analysis and biplot grouping with principal component analysis, can be employed to achieve this objective. These techniques allow for the grouping of genotypes based on agricultural, biochemical, and physiological traits, thereby facilitating the selection of diverse parents for crossbreeding programs and enhancing genetic diversity^61^. By utilizing these multivariate analysis techniques, distant groups of genotypes can be identified based on their traits, enabling their use as parents in crossbreeding programs to increase genetic diversity and potentially improve desirable traits in the offspring^62^.

The GSSR24 primer exhibited the highest PIC value in this study, indicating its efficacy in differentiating the studied populations of Sickleweed. These findings are consistent with a previous study by Piya et al.^29^, which also reported GSSR24 as having the highest PIC value in Sickleweed populations. Similar studies on other plants have shown high average PIC values for SSR markers, highlighting their effectiveness in assessing genetic diversity^23–27^. The GSSR24 primer also demonstrated the highest value of H, indicating its efficiency in distinguishing the studied populations. High values of genetic diversity reflect the marker's ability to differentiate genotypes from each other. The observed heterozygosity at certain loci may be attributed to gene introgression, microsatellite motif replication during the breeding season, or the evolutionary history of Sickleweed. The extent of genetic diversity is influenced by factors such as the type of molecular markers, characteristics of the SSR repeat unit, the number of SSR markers used, and the genetic relationships within the Sickleweed germplasm^24,25^. The effective polymorphism ratio, which represents the number of polymorphic loci in germplasm, varied between 2 for the ESSR80 marker and 4.8 for GSSR24. The marker's detection power (D), which reflects its ability to distinguish between two individuals, ranged from 0.781 for ESSR80 to 0.995 for GSSR24. These values indicate that the GSSR24 primer has a higher capacity for distinguishing between individuals. The variations in the number of alleles identified across different studies can be attributed to variations in the origin and characteristics of the studied genotypes, as well as differences in the markers and PCR conditions employed in each study^23,26,27^. PIC is an important criterion for comparing different markers in terms of their discriminatory power. Higher PIC values indicate greater polymorphism and the presence of rare alleles or alleles that significantly contribute to distinguishing individuals. Markers with high PIC values are particularly useful for distinguishing closely related genotypes^35–37^.

The primary objective of cluster analysis is to assess the degree of relatedness or genetic distance among populations. This approach enables researchers to reduce the time and effort required for random crossbreeding by strategically selecting distant populations from different clusters. By crossing populations that exhibit significant genetic distance, the chances of obtaining desired hybrids or achieving maximum segregation in subsequent generations, such as F_1_, can be enhanced^63^. Genetic diversity in plant species is influenced by various factors, including geographical distribution, population size, and breeding system^64,65^. It is essential to understand the genetic diversity of a plant species to plan and implement effective conservation strategies, irrespective of its geographical range^65^. A species' extensive geographic distribution does not necessarily ensure the preservation of its genetic diversity^64^. Therefore, conservation efforts should consider the genetic characteristics of the species. In many cases, the results obtained from molecular markers do not align perfectly with phenotypic traits. This discrepancy can be attributed to the polygenic control of agronomic traits and their susceptibility to environmental influences^33,36,37,66^. Morphological markers, which are based on agronomic data, may not accurately reflect the genetic differentiation of individuals based on their geographic environment. However, in this study, the agreement between cluster analysis using molecular markers and agronomic traits suggests that the chosen SSR markers provided sufficient coverage throughout the genome. Increasing the number of SSR markers could potentially yield even better separation within the studied germplasm, necessitating the design and utilization of additional SSR markers. Furthermore, since SSR markers are designed from non-coding regions of the genome, they may not target coding genes directly responsible for morphological traits. Therefore, the use of EST (Expressed Sequence Tag) markers, which are designed based on coding regions, is recommended for studying morphological traits and capturing a more comprehensive understanding of the genetic basis underlying these traits.

The results of the analysis of molecular variance (AMOVA) indicate that the variation within the sub-populations is greater than the variation between the sub-populations. This finding suggests that there is a wide range of allelic diversity within each population. Considering that the populations within each cluster are derived from different geographical locations, this result is consistent and highlights the significant diversity among the 15 populations studied^35,37^. It is important to note that increasing germplasm diversity leads to the presence of rare alleles within the population. While this can enhance the identification of potential associations, it also introduces challenges such as the increased likelihood of false relationships and a reduction in the statistical power of association mapping^67^. To ensure reliable results in association mapping studies, it is crucial to evaluate populations with high genetic and phenotypic diversity^68^. Despite the advantages of association mapping, it is important to acknowledge that the presence of population structure can potentially lead to false associations between markers and traits^69^.

The utilization of regression analysis to identify markers associated with traits can serve as an initial step for future studies focusing on QTL identification. The markers identified in this research, compared to other markers, may have a higher likelihood of being located in the coding regions of the studied traits. This is because they were included in the regression model and demonstrated a stronger association with the observed changes in those traits. Similar to other researchers in the field, regression analysis has been employed to establish the relationship between markers and traits in various plant species, ultimately facilitating plant breeding efforts^70–73^. The standardized β coefficients provide valuable information regarding the direction of the marker's effect on the trait. A negative sign indicates a reducing effect, while a positive sign suggests an increasing effect. Breeding programs can use genotypes lacking or possessing specific alleles identified by these markers to increase or decrease the expression of the target traits, aligning with the breeder's objectives. It is noteworthy that some markers were associated with multiple traits, indicating a close connection between these traits or potential pleiotropic control. This highlights the interdependence and shared genetic regulation among these traits, as observed in other studies^70,71^. The use of molecular markers related to important morphological traits in plant production and breeding, particularly through marker-assisted selection (MAS), allows for the identification of key genes and the introduction of candidate markers for further investigation in populations. While challenges exist, such as the scarcity of divergent populations for mapping and limitations in time and the correlation between morphological traits and molecular markers, regression analysis helps overcome these limitations and offers a promising approach for identifying markers associated with morphological traits.

In this study, it was observed that the population structure of Sickleweed was characterized by the presence of seven subgroups. However, these subgroups were not completely separated, indicating a significant degree of mixing and suggesting a mixed descent for the studied genotypes. This implies that individuals may inherit portions of their genome from different subgroups within the population. Additionally, the similarity in allele frequencies across different populations could be attributed to migration or shared descent^37,72^. The identification of markers associated with traits, as well as the relationship between certain markers and multiple traits, opens the door for further research in specific genomic regions. Validating these associated markers and converting them into specific SCAR markers or specific DNA sequences for targeted breeding is a practical and applicable solution. These markers can be utilized for important traits identified in this study^36,37^. The use of association analysis to identify markers linked to morphological traits in Sickleweed populations was documented for the first time in this study. The findings, along with previous research, suggest that highly associated and reliable markers for specific traits can be identified and utilized in future studies. However, it is important to employ larger and more diverse populations and incorporate a greater number of markers for more comprehensive investigations. These identified markers should be further examined in segregated populations and larger populations to confirm their correlation with specific traits. Ultimately, these markers can significantly enhance the effectiveness of breeding programs.

## Conclusion

The findings of this study highlight the high genetic diversity present in the examined Sickleweed population. The populations, originating from different regions and having distinct genetic backgrounds, exhibit variations in traits, indicating the presence of genetic factors influencing population differences alongside environmental effects. The use of SSR markers proves effective in studying the genetic diversity of the Sickleweed population, thanks to their stable positions in the genome. Selection based on molecular markers offers a rapid approach in breeding programs, with the genetic information obtained from these markers playing a crucial role. Thus, in addition to considering phenotypic traits, selecting superior genotypes and populations with high breeding value can be accomplished using molecular markers. It should be noted that the grouping of Sickleweed populations based on molecular data did not align closely with the grouping based on agricultural traits or the Bayesian method. However, the populations of Dillman-Gilan and Ilan-Qom exhibited the highest values for most nutritional elements traits. Considering the significance of these traits in human nutrition, these populations can be selected as superior populations and recommended for cultivation as vegetable sources for human consumption. Through stepwise regression analysis, 32 loci associated with the studied traits were identified. The results demonstrate that some markers are linked to multiple traits, underscoring their critical importance in plant breeding for simultaneously improving multiple traits.

## Data Availability

The data used to support the findings of this study are included in the article.
